# Epidemiological features and antibiotic resistance of *Salmonella* strains isolated from diarrheal cases in Gansu Province, China, 2024

**DOI:** 10.3389/fmicb.2026.1791129

**Published:** 2026-06-29

**Authors:** Guang Lan, Yanqin Shen, Jing Zhang, Baodi Li, Xianglai Sang, Dongli Liu

**Affiliations:** 1 Microbiology Laboratory, Gansu Provincial Center for Disease Control and Prevention, Lanzhou, China; 2 Institute of Pathogenic Microbiology, Shaanxi Provincial Center for Disease Control and Prevention, Xi’an, China

**Keywords:** antibiotic resistance, cgMLST, cgSNP, foodborne disease monitoring, multidrug resistance, *Salmonella*

## Abstract

**Introduction:**

*Salmonella* is a significant global health threat, particularly in developing areas. This study elucidated the epidemiological features, serotypes, antibiotic resistance patterns, and molecular evolutionary relationships of 91 *Salmonella* strains isolated from diarrhea cases in Gansu Province, China, in 2024.

**Methods:**

Antibiotic susceptibility was assessed using the broth microdilution method. Whole-genome sequencing and bioinformatics analyses were performed to determine serotype, genetic relationships, antimicrobial resistance gene prevalence, and virulence gene profiles.

**Results:**

Among the 91 strains, the majority (56.04%, 51/91) were isolated from children aged 0–9 years, indicating this group as a high-risk population. Of the strains, 41.76% (38/91) originated from Lanzhou City and 17.59% (16/91) from Qingyang. In total, 20 distinct serotypes were identified, with monophasic *Salmonella Typhimurium* serovar 1,4,[5],12:i:- being the most prevalent at 34.07% (31/91), followed by serovar Enteritidis at 25.27% (23/91). Genetic diversity revealed 22 sequence types corresponding to these serotypes. The antibiotic resistance profiles indicated high resistance rates (>50%) to several antibiotics: streptomycin (69.0%), ampicillin (78.0%), cefazolin (58.0%), tetracycline (55.0%), and ampicillin/sulbactam (55.0%). Notably, 80.22% (73/91) of the strains exhibited multidrug resistance. Substantial serotype-specific differences in resistance gene profiles were observed: serotype 1,4,[5],12:i:- carried high frequencies of heavy metal resistance genes (e.g., silver, 83.9%), while serotype London uniquely harbored quaternary ammonium disinfectant resistance genes (71.4%). Quinolone resistance genes had the highest detection rate (81.32%), followed by streptomycin (70.33%) and sulfonamide (68.13%) resistance genes. Formal testing of genotype-phenotype association revealed a weak and non-significant correlation (Spearman ρ = 0.14, *p* > 0.05), indicating that resistance gene carriage alone does not fully predict phenotypic resistance. Phylogenetic analysis based on core genome multilocus sequence typing identified 15 minimum spanning tree clusters, with the largest cluster comprising ST11 *Salmonella enterica*. Additionally, isolates from Lanzhou and Baiyin City shared identical single nucleotide polymorphism characteristics, suggesting a potential risk of inter-regional transmission. Virulence gene analysis confirmed that all strains possessed intact SPI-1/SPI-2 pathogenicity islands (carriage rates 92.7–98.5%) and lacked Vi capsule genes, consistent with their non-typhoidal status and diarrheal presentation.

**Discussion:**

This study reveals an alarming multidrug resistance rate, predominance of specific serotypes, and potential inter-regional transmission of epidemic clones in Gansu Province, underscoring the urgent need for enhanced genomic surveillance and targeted interventions.

## 1 Introduction

*Salmonella* is a genus of Gram-negative bacteria that is widely distributed in nature and belongs to the Enterobacteriaceae family. It is characterized by flagellar motility and facultative anaerobic metabolism, demonstrating remarkable environmental adaptability. Moreover, it has over 2600 serovars with varying host specificities and virulence profiles (Bai et al., 2022). It can lead to a range of health issues ranging from self-limiting gastroenteritis to severe systemic complications, such as bacteremia, reactive arthritis, and life-threatening septic shock, particularly threatening immunocompromised individuals, children under five, and the elderly (GBD 2017 Non-Typhoidal Salmonella Invasive Disease Collaborators, 2019; Clinical and Laboratory Standards Institute [CLSI], 2024). Notably, *Salmonella* is one of the four key global causes of diarrhoeal diseases (Centers for Disease Control and Prevention, 2025; World Health Organization, 2025b). The transmission of *Salmonella* primarily occurs through the fecal-oral route via contaminated food products, particularly poultry, eggs, and fresh produce, as well as through contaminated water sources. Factors such as inadequate infrastructure, limited access to clean water, and improper food handling practices play pivotal roles in perpetuating health challenges related to *Salmonella* infections. Therefore, controlling *Salmonella* transmission is essential for effective food safety systems and public health infrastructure.

In developing countries, *Salmonella* infections are particularly prevalent due to substandard sanitary conditions and insufficient food safety regulations (Odeyemi and Bamidele, 2016; Li et al., 2022). Contaminated poultry meat serves as a primary conduit for foodborne transmission of *Salmonella* to humans, significantly contributing to the high incidence of these infections (Butaye et al., 2006). A 2022 meta-analysis indicated that the overall average prevalence of *Salmonella* was higher in northern China compared to southern China (Chen et al., 2024). Furthermore, economic instability often results in limited resources for health education, enforcement of food safety standards, and timely medical interventions, thereby exacerbating the risk of *Salmonella* outbreaks (Ayachi et al., 2009).

Gansu Province, located in the northwest region of China, presents unique geographical and climatic conditions, along with distinct agricultural and pastoral production methods, which may influence the distribution and epidemiological characteristics of *Salmonella*. The province is characterized by diverse climates ranging from arid to semi-arid, which affects food production and storage methods. Additionally, the unique agricultural and pastoral production methods, combining traditional practices with modern farming techniques, may either limit or facilitate the spread of pathogens, including *Salmonella* (Li et al., 2023). Understanding these factors is essential for determining the epidemiological patterns of *Salmonella* in this region.

Despite its significance, research on the whole-genome features of *Salmonella* in Gansu Province remains limited (Liu and Zeng, 2024). This scarcity of comprehensive studies restricts the development of effective prevention and control strategies for *Salmonella* infections in the region. Therefore, this study aims to analyze the characteristics of 91 *Salmonella* strains isolated from diarrhea cases in Gansu Province in 2024, revealing their epidemiological features, resistance patterns, and molecular evolutionary relationships. Our findings may provide a scientific foundation for the prevention and control of *Salmonella* infections both in Gansu Province and nationwide.

## 2 Materials and methods

### 2.1 Ethics statement

This study involved only bacterial strains isolated from routine diagnostic specimens. The strains were anonymized and not linked to any identifiable patient information. According to the guidelines of the Ethics Committee of Gansu Provincial Center for Disease Control and Prevention, research using only anonymized bacterial strains without any patient data is considered exempt from ethical review. Therefore, ethical approval was not required for this study.

### 2.2 Sample source

This study analyzed a total of 91 strains of *Salmonella*, which were collected from patients presenting with diarrhea at sentinel hospitals across various regions of Gansu Province in 2024. Diarrhea was defined as three or more loose or watery stools within a 24-h period. Sampling covered nine prefecture-level cities (Lanzhou, Qingyang, Baiyin, Tianshui, Jiuquan, Wuwei, Pingliang, Gannan Prefecture, and Zhangye) with proportional allocation by population to ensure representativeness. The isolates were stratified by patient age (0–9, 10–19, 20–29, 30–39, 40–49, 50–59, 60–69, 70–79, and ≥80 years), gender (male and female), geographic region (by prefecture-level city), and month of isolation (January to December 2024). The basic information is detailed in the Supplementary Table 1. The sampling process involved collaboration with healthcare professionals in these hospitals to ensure accurate identification of cases suspected of being caused by *Salmonella*. For strain isolation, all samples were processed following established protocols outlined in the “National Foodborne Disease Surveillance Work Manual—Standard Operating Procedures for Fecal Specimen Examination.” After isolation, the strains were subjected to serological and biochemical testing to confirm their identity as *Salmonella*.

### 2.3 Antibiotic susceptibility testing

Antibiotic susceptibility testing was conducted under the “Standard Operating Procedures for Antibiotic Susceptibility Testing of Foodborne Pathogenic Bacteria.” The microdilution method was used for this testing, as recommended by the Clinical and Laboratory Standards Institute (CLSI) M100-S34 guidelines (Clinical and Laboratory Standards Institute [CLSI], 2024). The resistance to 29 antibiotics across 11 major categories, which included classes such as β-lactams, aminoglycosides, tetracyclines, and fluoroquinolones, among others, was tested. The selection of antibiotics tested was guided by a review of commonly used antibiotics from Chinese and international resistance monitoring networks, as well as an analysis of previous resistance monitoring data. To ensure the accuracy and reliability of the resistance results, *Pseudomonas aeruginosa* ATCC 27853 served as a quality control strain. The minimal inhibitory concentration (MIC) was established for each *Salmonella* strain against the selected antibiotics. Based on the MIC values and corresponding interpretation standards, the susceptibility of each tested strain to the antibiotic was reported as sensitive, intermediate, or resistant.

### 2.4 Whole genome sequencing

Whole genome sequencing was commissioned and performed by Shaanxi Yunshen Pharmaceutical Technology Co., Ltd (Xi’an, China). Briefly, the genomic DNA of *Salmonella* was extracted using a bacterial genomic DNA extraction kit (RocGene, Beijing, China). Whole genome sequencing was conducted on the GenoLab M platform (GeneMind Biosciences, Shenzhen, China) using the PE150 mode. Paired-end sequencing libraries were constructed. To obtain high-coverage genomic sequence data, the sequencing read length was required to be at least 150 bp, with high-quality data (referred to as “Clean data”) amounting to at least 1 G. The genome coverage was set at a minimum of 95%, while the coverage of gene regions was required to be at least 98%. The overall coverage depth was determined to be a minimum of 100×. Additionally, the base data quality values had to meet the following criteria: Q20 ≥ 95% and Q30 ≥ 85%. The number of scaffolds was limited to fewer than 100 and the number of contigs to fewer than 200, with a single-base error rate maintained at less than 1 in 100,000.

### 2.5 Raw sequencing data processing and bioinformatics analysis

FastQC version 0.12.1^1^ was used for the quality control of raw sequencing data, while SPAdes version 3.15.4^2^ facilitated bacterial genome assembly. AMRFinderPlus software version 3.11.26 was employed to predict antimicrobial resistance genes (Feldgarden et al., 2019). Additionally, the *Salmonella In Silico* Typing Resource (SISTR) software version 1.1.1 was utilized for serotype predictions (Yoshida et al., 2016). Ridom SeqSphere+ (Version 10.0.5) software (Ridom GmbH, Germany) (Junemann et al., 2013) was applied for multilocus sequence typing (MLST) and core genome MLST (cgMLST) to construct a minimum spanning tree. For selected clustered strains, core genome single nucleotide polymorphism (cgSNP) analysis was performed using Ridom SeqSphere+ software (Version 10.0.5) to develop a phylogenetic tree for the *Salmonella* genus, elucidating molecular evolutionary patterns and population differentiation characteristics. Virulence genes were predicted using the *Salmonella enterica* VFDB analysis workflow implemented in Ridom SeqSphere+ software (Chen et al., 2016), screening against the VFDB database.^3^

### 2.6 Statistical analysis

Categorical variables were compared using Chi-square test or Fisher’s exact test. Resistance gene differences were analyzed by Kruskal-Wallis H test with Bonferroni correction. Genotype–phenotype associations were evaluated using Spearman rank correlation coefficient test. *P* < 0.05 was considered statistically significant.

## 3 Results

### 3.1 Strain distribution

**FIGURE 1:**
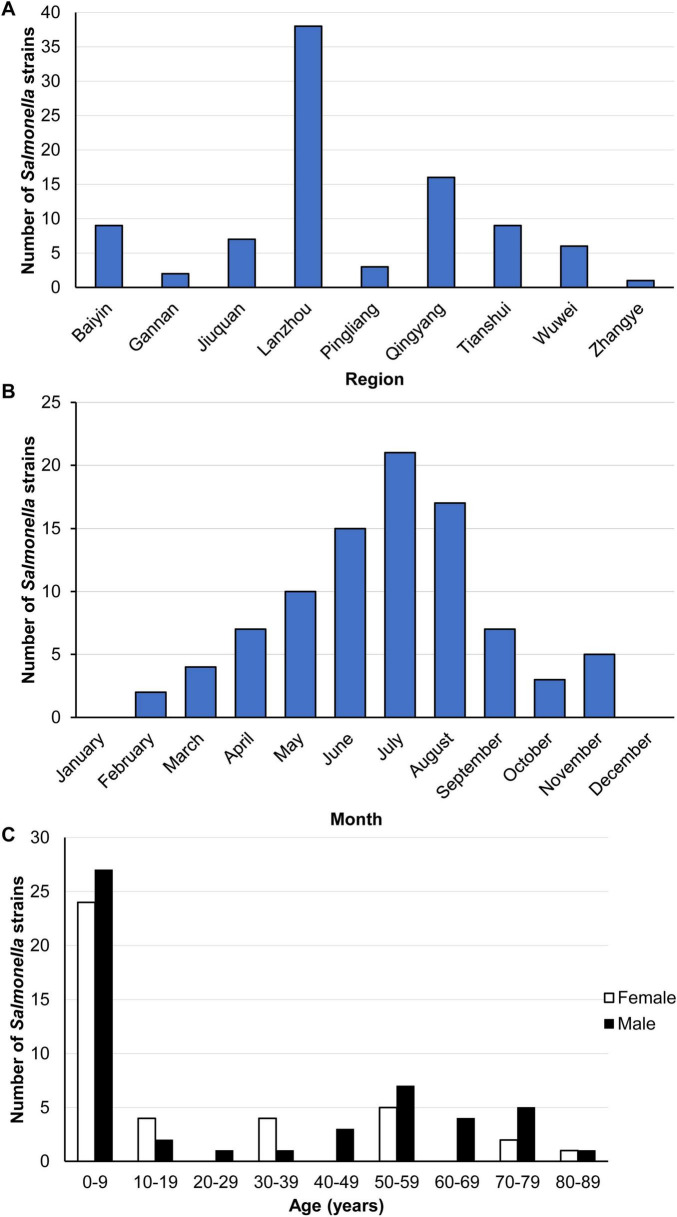
Distribution characteristics of 91 *Salmonella* strains in Gansu Province, 2024. **(A)** Geographic distribution. Bars show the number of isolates from each of the nine prefecture-level cities. **(B)** Monthly distribution. Bars show the number of isolates per month from January to December. **(C)** Age and gender distribution. Bars show the number of isolates by age group (0–9 to ≥80 years).

A total of 91 strains were collected from 9 prefecture-level cities, with Lanzhou having the highest number (38 strains, 41.76%), followed by Qingyang (16, 17.59%), Baiyin (9, 9.89%), Tianshui (9, 9.89%), Jiuquan (7, 7.69%), Wuwei (6, 6.59%), Pingliang (3, 3.30%), Gannan Prefecture (2, 2.20%), and Zhangye (1, 1.10%) (Figure 1A). There were statistically significant differences in the geographic distribution of isolates (*P* < 0.05), indicating uneven distribution across cities and suggesting potential regional clustering.

The monthly distribution of isolates was as follows: January (0/91, 0%), February (2/91, 2.20%), March (4/91, 4.40%), April (7/91, 7.69%), May (10/91, 10.99%), June (15/91, 16.48%), July (21/91, 23.08%), August (17/91, 18.68%), September (7/91, 7.69%), October (3/91, 3.30%), November (5/91, 5.49%), and December (0/91, 0%) (Figure 1B). The majority of strains were concentrated in summer (June–August), accounting for 58.24% (53/91) of the total, which is consistent with the high-incidence season of foodborne diseases in northwest China.

In terms of demographic distribution, significant differences were observed across age groups (χ^2^ = 194.330, *P* < 0.001), primarily due to the overrepresentation of children aged 0–9 years (51/91, 56.04%) (Figure 1C), indicating that children are a high-risk population for *Salmonella* infection. Among the isolates, 51 (56.04%) were from males and 40 (43.96%) from females, yielding a male-to-female ratio of 1.27:1. There was no statistically significant difference in the overall gender distribution (χ^2^ = 1.330, *P* > 0.05). For age groups with sufficient sample sizes (≥2 isolates per gender), no significant gender differences were detected. Within the 0–9-year age group, the gender ratio was balanced (χ^2^ = 0.177, *P* > 0.05). In age groups with small sample sizes (e.g., 20–29, 40–49, and 60–69 years containing only male isolates with 1–4 cases), statistical analysis was not feasible.

### 3.2 Genetic serotyping and sequence typing

**FIGURE 2:**
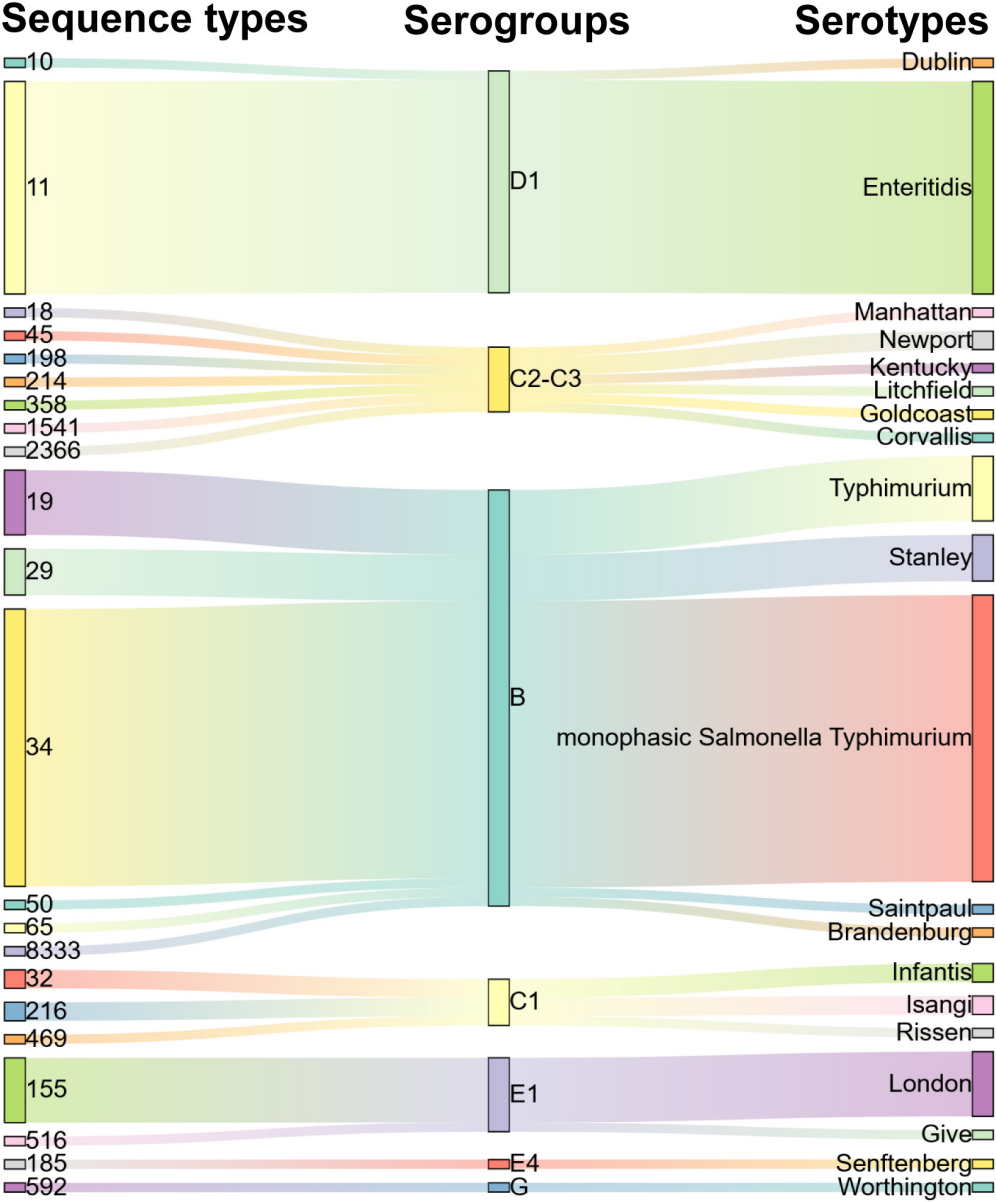
The serotypes, serogroups, and sequence types of 91 strains of *Salmonella* in Gansu Province.

A total of 91 strains of *Salmonella* were identified through gene sequencing, revealing seven serogroups, 20 serotypes, and 22 sequence types (STs), as illustrated in Figure 2. The distribution characteristics of the dominant serotypes were as follows: serovar 1,4,[5],12:i:− exhibited the highest detection rate at 34.07% (31/91), followed by serovar Enteritidis at 25.27% (23/91). Both serovar London and Typhimurium represented 7.69% (7 cases each), while serovar Stanley accounted for 5.49% (five cases). Associations were observed among serogroups, serotypes, and STs. Serovar Enteritidis predominantly corresponded to ST11 and serogroup D1, whereas serovar 1,4,[5],12:i:− was closely related to ST34 and serogroup B. Geographically, serovar Enteritidis and 1,4,[5],12:i:− were the dominant strains in Lanzhou. Temporal analysis revealed a higher detection rate for serovar Enteritidis in August. Gender-wise, both dominant serotypes maintained a leading presence in both male and female populations. Notably, in infants under 1 year old, serovar 1,4,[5],12:i:− was predominant, accounting for 58.33%, while serovar Enteritidis demonstrated a widespread distribution across various age groups. This predominance is clinically concerning, as this serotype exhibited the highest multidrug resistance rate (90.32%, 28/31) among all serotypes identified in this study, potentially complicating empirical antibiotic treatment in this vulnerable population.

### 3.3 Antimicrobial resistance and sensitivity profiles of *Salmonella* strains

Antibiotic susceptibility testing indicated that the *Salmonella* strains showed high resistance (resistance rate > 50%) to streptomycin (69.0%), ampicillin (78.0%), cefazolin (58.0%), tetracycline (55.0%), and ampicillin/sulbactam (55.0%) (Table 1). In contrast, the *Salmonella* strains presented low resistance (resistance rate < 10%) to imipenem (10.0%), ertapenem (7.0%), amikacin (1.0%), and tigecycline (1.0%). Additionally, the *Salmonella* strains displayed significant intermediate resistance (resistance rate > 20%) to cefazolin (25.0%), amoxicillin/clavulanate (26.0%), and ciprofloxacin (44.0%). Notably, the *Salmonella* strains demonstrated high sensitivity (sensitivity rate > 80%) to ertapenem (81.0%), imipenem (84.0%), tigecycline (98.0%), and amikacin (98.0%). These findings suggest that, in clinical settings, it is prudent to avoid the use of highly resistant antibiotics, such as ampicillin and streptomycin, and to consider more sensitive alternatives, such as tigecycline and amikacin.

**TABLE 1:** Antimicrobial resistance and sensitivity profiles of 91 *Salmonella* strains in Gansu Province.

Antibiotic category	Antibiotic	Resistance rate (%)	Intermediate rate (%)	Sensitivity rate (%)
β-lactam derivatives (penicillins)	Ampicillin	78% (71/91)	3% (3/91)	19% (17/91)
β-lactam compounds (carbapenems)	Ertapenem	7% (6/91)	12% (11/91)	81% (74/91)
β-lactam compounds (carbapenems)	Imipenem	10% (9/91)	7% (6/91)	84% (76/91)
β-lactam compounds (carbapenems)	Meropenem	14% (13/91)	9% (8/91)	77% (70/91)
β-lactam (cephalosporins)	Ceftazidime	23% (21/91)	3% (3/91)	74% (67/91)
β-lactam (cephalosporins)	Ceftiofur	30% (27/91)	1% (1/91)	69% (63/91)
β-lactam (cephalosporins)	Cefoxitin	14% (13/91)	5% (5/91)	80% (73/91)
β-lactam (cephalosporins)	Cefazolin	58% (53/91)	25% (23/91)	16% (15/91)
β-lactam (cephalosporins)	Cefepime	19% (17/91)	7% (6/91)	75% (68/91)
β-lactam (cephalosporins)	Cefotaxime	29% (26/91)	1% (1/91)	70% (64/91)
β-lactam (cephalosporins)	Cefuroxime	37% (34/91)	1% (1/91)	62% (56/91)
Combination of β-lactam and enzyme inhibitors	Amoxicillin/clavulanate	23% (21/91)	26% (24/91)	51% (46/91)
Combination of β-lactam and enzyme inhibitors	Ampicillin/sulbactam	55% (50/91)	21% (19/91)	24% (22/91)
Combination of β-lactam and enzyme inhibitors	Ceftazidime/clavulanic acid	12% (11/91)	2% (2/91)	86% (78/91)
Combination of β-lactam and enzyme inhibitors	Cefotaxime/clavulanic acid	16% (15/91)	0% (0/91)	84% (76/91)
Combination of β-lactam and enzyme inhibitors	Ceftazidime/avibactam	13% (12/91)	0% (0/91)	87% (79/91)
Aminoglycosides	Amikacin	1% (1/91)	1% (1/91)	98% (89/91)
Aminoglycosides	Gentamicin	21% (19/91)	1% (1/91)	78% (71/91)
Aminoglycosides	Streptomycin	69% (63/91)	0% (0/91)	31% (28/91)
Macrolides	Azithromycin	13% (12/91)	0% (0/91)	86% (78/91)
Sulfonamide/trimethoprim combination	Sulfamethoxazole/trimethoprim	33% (30/91)	0% (0/91)	66% (60/91)
Quinolones	Ciprofloxacin	23% (21/91)	44% (40/91)	33% (30/91)
Quinolones	Naphthidine acid	44% (40/91)	0% (0/91)	56% (51/91)
*Lactobacillus* peptide lipopeptides	Polymyxin E	18% (16/91)	0% (0/91)	81% (74/91)
*Lactobacillus* peptide lipopeptides	Polymyxin B	19% (17/91)	0% (0/91)	81% (74/91)
Tetracyclines	Tigecycline	1% (1/91)	1% (1/91)	98% (89/91)
Tetracyclines	Tetracycline	55% (50/91)	1% (1/91)	44% (40/91)
Amide alcohols	Chloramphenicol	40% (36/91)	1% (1/91)	59% (54/91)
Amide alcohols	Florfenicol	38% (35/91)	7% (6/91)	55% (50/91)

### 3.4 Prevalence and patterns of multidrug resistance among *Salmonella* strains

A total of 73 *Salmonella* strains exhibited resistance to three or more categories of antimicrobial agents, yielding a multidrug resistance rate of 80.22% (73/91) (Table 2). Among these multidrug resistance strains, 45 distinct resistance profiles (i.e., combinations of antibiotic classes) were identified. The most prevalent profile, accounting for 8.22% (6/73) of the multidrug resistance strains, comprised concurrent resistance to β-lactams (penicillins), aminoglycosides, and tetracyclines. Notably, three strains demonstrated extensive drug resistance, with resistance spanning 10–11 antibiotic classes. The most complex profile encompassed resistance to all β-lactam subclasses (penicillins, cephalosporins, carbapenems, monobactams, and β-lactamase inhibitor combinations) along with several other major categories (Table 2).

**TABLE 2:** Prevalence and patterns of multidrug resistance among 91 *Salmonella* strains in Gansu Province.

Number of antibiotics	Multidrug-resistant antibiotic categories	Number of strains	Percentage of strains
11	Beta-lactams (carbapenems); beta-lactams (cephalosporins); amides alcohols; tetracyclines; sulfonamides; aminoglycosides; beta-lactam/enzyme inhibitor combinations; beta-lactams (penicillins); quinolones; glycylcyclines; macrolides	1	1.37%
10	Beta-lactams (carbapenems); beta-lactams (cephalosporins); amides alcohols; tetracyclines; sulfonamides; aminoglycosides; beta-lactam/enzyme inhibitor combinations; beta-lactams (penicillins); quinolones; macrolides	2	2.74%
9	Beta-lactams (carbapenems); beta-lactams (cephalosporins); amides alcohols; tetracyclines; sulfonamides; aminoglycosides; beta-lactam/enzyme inhibitor combinations; beta-lactams (penicillins); quinolones	2	2.74%
9	Beta-lactams (cephalosporins); amides alcohols; tetracyclines; sulfonamides; aminoglycosides; beta-lactam/enzyme inhibitor combinations; beta-lactams (penicillins); quinolones; macrolides	2	2.74%
9	Beta-lactams (carbapenems); beta-lactams (cephalosporins); amides alcohols; tetracyclines; sulfonamides; aminoglycosides; beta-lactam/enzyme inhibitor combinations; beta-lactams (penicillins); macrolides	1	1.37%
9	Beta-lactams (cephalosporins); amides alcohols; sulfonamides; aminoglycosides; beta-lactam/enzyme inhibitor combinations; beta-lactams (penicillins); quinolones; bacteriocin lipopeptides; macrolides	1	1.37%
8	Beta-lactams (cephalosporins); amides alcohols; tetracyclines; sulfonamides; aminoglycosides; beta-lactam/enzyme inhibitor combinations; beta-lactams (penicillins); quinolones	3	4.11%
8	Beta-lactams (carbapenems); beta-lactams (cephalosporins); amides alcohols; tetracyclines; sulfonamides; aminoglycosides; beta-lactam/enzyme inhibitor combinations; beta-lactams (penicillins)	1	1.37%
8	Beta-lactams (cephalosporins); amides alcohols; tetracyclines; sulfonamides; aminoglycosides; beta-lactam/enzyme inhibitor combinations; beta-lactams (penicillins); macrolides	1	1.37%
8	Amides alcohols; tetracyclines; sulfonamides; aminoglycosides; beta-lactam/enzyme inhibitor combinations; beta-lactams (penicillins); quinolones; macrolides	1	1.37%
7	Beta-lactams (carbapenems); beta-lactams (cephalosporins); aminoglycosides; beta-lactam/enzyme inhibitor combinations; beta-lactams (penicillins); quinolones; bacteriocin lipopeptides	4	5.48%
7	Beta-lactams (cephalosporins); amides alcohols; tetracyclines; sulfonamides; aminoglycosides; beta-lactam/enzyme inhibitor combinations; beta-lactams (penicillins)	4	5.48%
7	Beta-lactams (cephalosporins); amides alcohols; tetracyclines; aminoglycosides; beta-lactam/enzyme inhibitor combinations; beta-lactams (penicillins); quinolones	2	2.74%
7	Beta-lactams (cephalosporins); tetracyclines; aminoglycosides; beta-lactam/enzyme inhibitor combinations; beta-lactams (penicillins); quinolones; bacteriocin lipopeptides	1	1.37%
7	Beta-lactams (cephalosporins); amides alcohols; sulfonamides; aminoglycosides; beta-lactam/enzyme inhibitor combinations; beta-lactams (penicillins); macrolides	1	1.37%
7	Beta-lactams (cephalosporins); amides alcohols; tetracyclines; sulfonamides; aminoglycosides; beta-lactams (penicillins); quinolones	1	1.37%
7	Amides alcohols; tetracyclines; sulfonamides; aminoglycosides; beta-lactam/enzyme inhibitor combinations; beta-lactams (penicillins); quinolones	1	1.37%
6	Beta-lactams (cephalosporins); amides alcohols; tetracyclines; aminoglycosides; beta-lactam/enzyme inhibitor combinations; beta-lactams (penicillins)	4	5.48%
6	Beta-lactams (cephalosporins); tetracyclines; aminoglycosides; beta-lactam/enzyme inhibitor combinations; beta-lactams (penicillins); quinolones	3	4.11%
6	Amides alcohols; tetracyclines; sulfonamides; aminoglycosides; beta-lactam/enzyme inhibitor combinations; beta-lactams (penicillins)	2	2.74%
6	Beta-lactams (carbapenems); beta-lactams (cephalosporins); tetracyclines; aminoglycosides; beta-lactam/enzyme inhibitor combinations; bacteriocin lipopeptides	1	1.37%
6	Beta-lactams (cephalosporins); aminoglycosides; beta-lactam/enzyme inhibitor combinations; beta-lactams (penicillins); quinolones; bacteriocin lipopeptides	1	1.37%
6	Beta-lactams (cephalosporins); tetracyclines; sulfonamides; aminoglycosides; beta-lactam/enzyme inhibitor combinations; beta-lactams (penicillins)	1	1.37%
6	Beta-lactams (cephalosporins); amides alcohols; tetracyclines; aminoglycosides; beta-lactams (penicillins); quinolones	1	1.37%
6	Beta-lactams (cephalosporins); amides alcohols; tetracyclines; sulfonamides; aminoglycosides; beta-lactam/enzyme inhibitor combinations	1	1.37%
5	Beta-lactams (cephalosporins); aminoglycosides; beta-lactam/enzyme inhibitor combinations; beta-lactams (penicillins); quinolones	3	4.11%
5	Beta-lactams (carbapenems); sulfonamides; beta-lactams (penicillins); quinolones; bacteriocin lipopeptides	1	1.37%
5	Beta-lactams (cephalosporins); beta-lactam/enzyme inhibitor combinations; beta-lactams (penicillins); quinolones; macrolides	1	1.37%
5	Beta-lactams (cephalosporins); tetracyclines; beta-lactams (penicillins); quinolones; bacteriocin lipopeptides	1	1.37%
5	Beta-lactams (cephalosporins); tetracyclines; beta-lactam/enzyme inhibitor combinations; beta-lactams (penicillins); quinolones	1	1.37%
5	Tetracyclines; aminoglycosides; beta-lactam/enzyme inhibitor combinations; beta-lactams (penicillins); quinolones	1	1.37%
5	Amides alcohols; sulfonamides; aminoglycosides; beta-lactam/enzyme inhibitor combinations; beta-lactams (penicillins)	1	1.37%
5	Amides alcohols; tetracyclines; aminoglycosides; beta-lactams (penicillins); quinolones	1	1.37%
4	Beta-lactams (cephalosporins); beta-lactams (penicillins); quinolones; bacteriocin lipopeptides	2	2.74%
4	Beta-lactams (cephalosporins); beta-lactam/enzyme inhibitor combinations; beta-lactams (penicillins); quinolones	2	2.74%
4	Tetracyclines; aminoglycosides; β-lactams (penicillins); quinolones	2	2.74%
4	β-lactams (carbapenems); tetracyclines; aminoglycosides; β-lactam/β-lactamase inhibitor combinations	1	1.37%
4	β-lactams (cephalosporins); aminoglycosides; β-lactam/β-lactamase inhibitor combinations; β-lactams (penicillins)	1	1.37%
4	Aminoglycosides; β-lactam/β-lactamase inhibitor combinations; β-lactams (penicillins); quinolones	1	1.37%
4	Amphenicols; tetracyclines; sulfonamides; macrolides	1	1.37%
3	Tetracyclines; aminoglycosides; β-lactams (penicillins)	6	8.22%
3	β-lactams (cephalosporins); β-lactams (penicillins); quinolones	1	1.37%
3	β-lactams (cephalosporins); β-lactam/β-lactamase inhibitor combinations; lantibiotics	1	1.37%
3	β-lactams (cephalosporins); aminoglycosides; β-lactams (penicillins)	1	1.37%
3	Sulfonamides; β-lactams (penicillins); quinolones	1	1.37%

Analysis of the antibiotic class composition within the multidrug resistance profiles revealed that β-lactams (penicillins) were the most frequently involved (93.2%, 68/73), followed by aminoglycosides (83.56%, 61/73). Other commonly co-occurring resistance classes included β-lactam/β-lactamase inhibitor combinations (73.97%, 54/73), β-lactams (cephalosporins) (72.60%, 53/73), tetracyclines (68.49%, 50/73), quinolones (60.27%, 44/73), phenicols (47.95%, 35/73), and sulfonamides (41.10%, 30/73), collectively forming the core framework of multidrug resistance observed.

**FIGURE 3:**
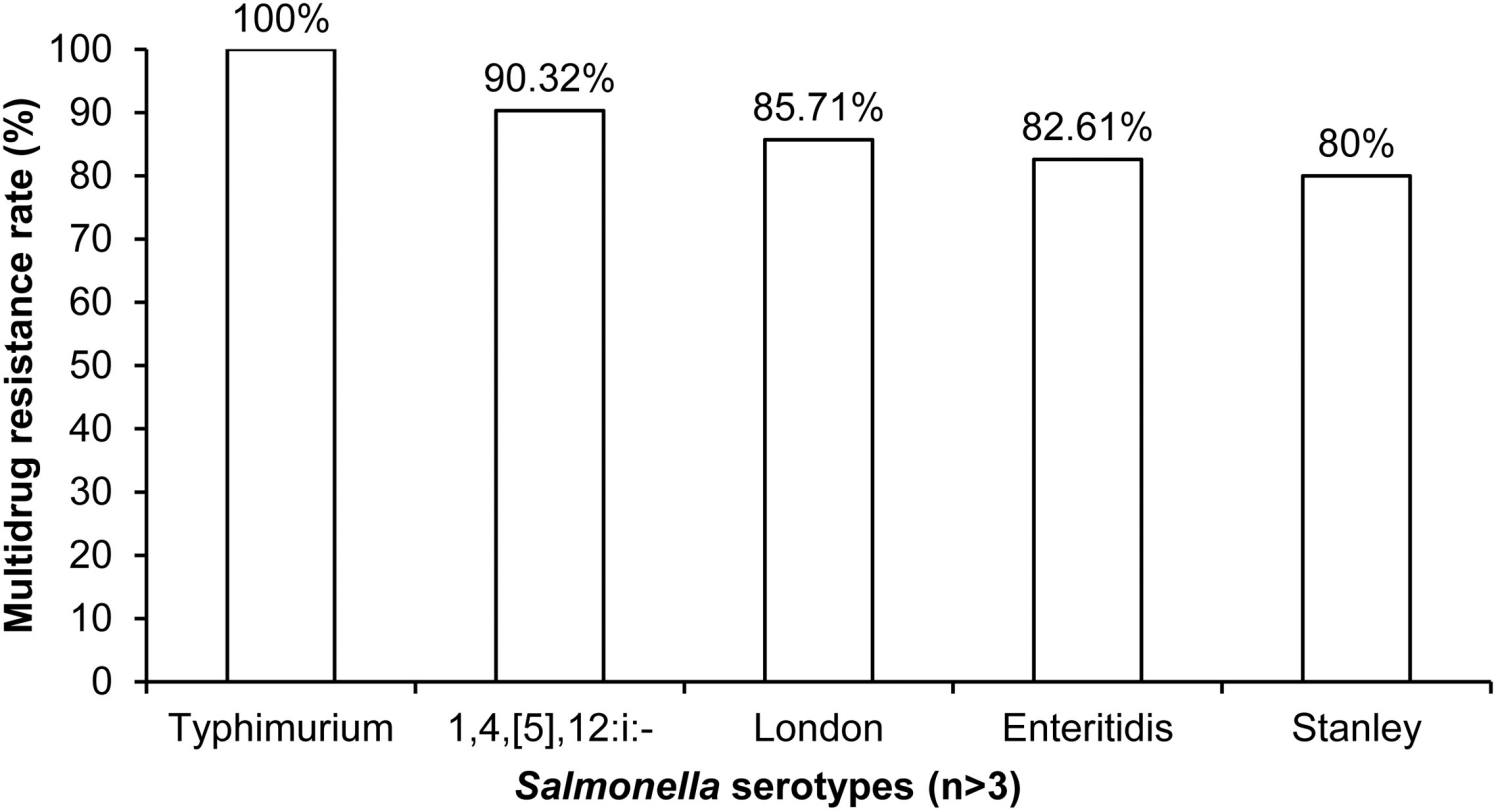
Multidrug resistance rates of *Salmonella* serotypes (*n* > 3).

The multidrug resistance rates varied among the dominant serotypes: serotype 1,4,[5],12:i:− (90.32%, 28/31), Enteritidis (82.61%, 19/23), Typhimurium (100.00%, 7/7), and London (85.71%, 6/7) (Figure 3). However, a chi-square test revealed no statistically significant difference in multidrug resistance rates among these serotypes (χ^2^ = 2.214, *P* = 0.696). Pairwise comparisons using Fisher’s exact test also showed no significant differences between any two serotypes (all *P* > 0.05). Resistance to 10 antibiotic classes was observed in one strain each of serotypes Stanley and Kentucky, while resistance to 11 classes was identified in one strain of serotype 1,4,[5],12:i:−.

### 3.5 Prevalence and distribution of antimicrobial resistance genes in *Salmonella* strains

The analysis of antimicrobial resistance genes revealed significant differences in resistance gene carriage rates across functional categories, as indicated by the Kruskal-Wallis test results (H = 609.2, *p* < 0.0001). The highest carriage rates were observed for efflux pumps, heavy metal resistance, and virulence/stress genes, each at 100%. The lowest carriage rates of antimicrobial resistance genes were observed for polymyxins (28.57%), glycopeptides (28.57%), and macrolides (42.86%). The most critical significant pairs, as determined by Bonferroni correction, included efflux pumps/heavy metals/virulence versus polymyxins (*p* = 1.98e−39), macrolides versus quinolones (*p* = 1.18e−17), and beta-lactams versus sulfonamides (*p* = 1.51e−5).

**FIGURE 4:**
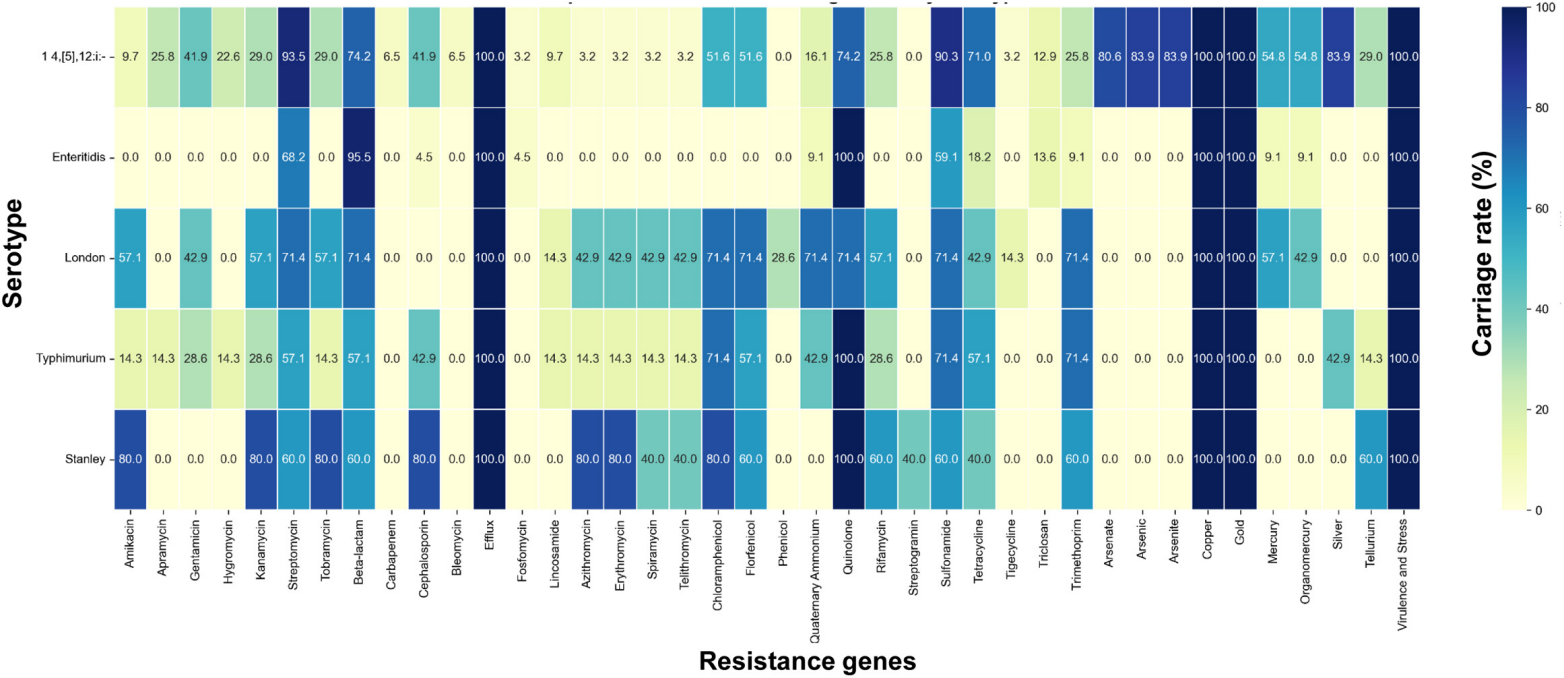
Heatmap of antimicrobial resistance gene distribution among major *Salmonella* serotypes. The heatmap shows the prevalence (%) of antimicrobial resistance genes across five major serotypes: 1,4,[5],12:i:– (*n* = 31), Enteritidis (*n* = 23), Typhimurium (*n* = 7), London (*n* = 7), and Stanley (*n* = 5). Colors represent carriage rates ranging from 0% (light yellow) to 100% (dark blue). Resistance genes are grouped by antibiotic class on the y-axis.

Among the five major serotypes, notable differences in resistance gene profiles were observed (Figure 4). Serotype 1,4,[5],12:i:− exhibited the most complex resistance profile, with high frequencies of heavy metal resistance genes including arsenite (83.9%), arsenate (80.6%), and silver (83.9%), as well as antibiotic resistance genes such as tetracycline (71.0%) and mercury (54.8%). This serotype may originate from heavy metal-contaminated environments. In contrast, serotype Enteritidis demonstrated a relatively lower resistance burden, primarily carrying core resistance genes for beta-lactams (95.5%), streptomycin (68.2%), and sulfonamides (59.1%). Serotype London specifically carried quaternary ammonium disinfectant resistance genes (71.4%), suggesting potential exposure to disinfectant-rich environments, with high frequencies of amikacin (57.1%) and mercury (57.1%). Serotype Stanley exhibited broad resistance to aminoglycosides and macrolides, including amikacin (80%) and kanamycin (80%). Serotype Typhimurium showed relatively high carriage rates of trimethoprim (71.4%) and chloramphenicol (71.4%).

**FIGURE 5:**
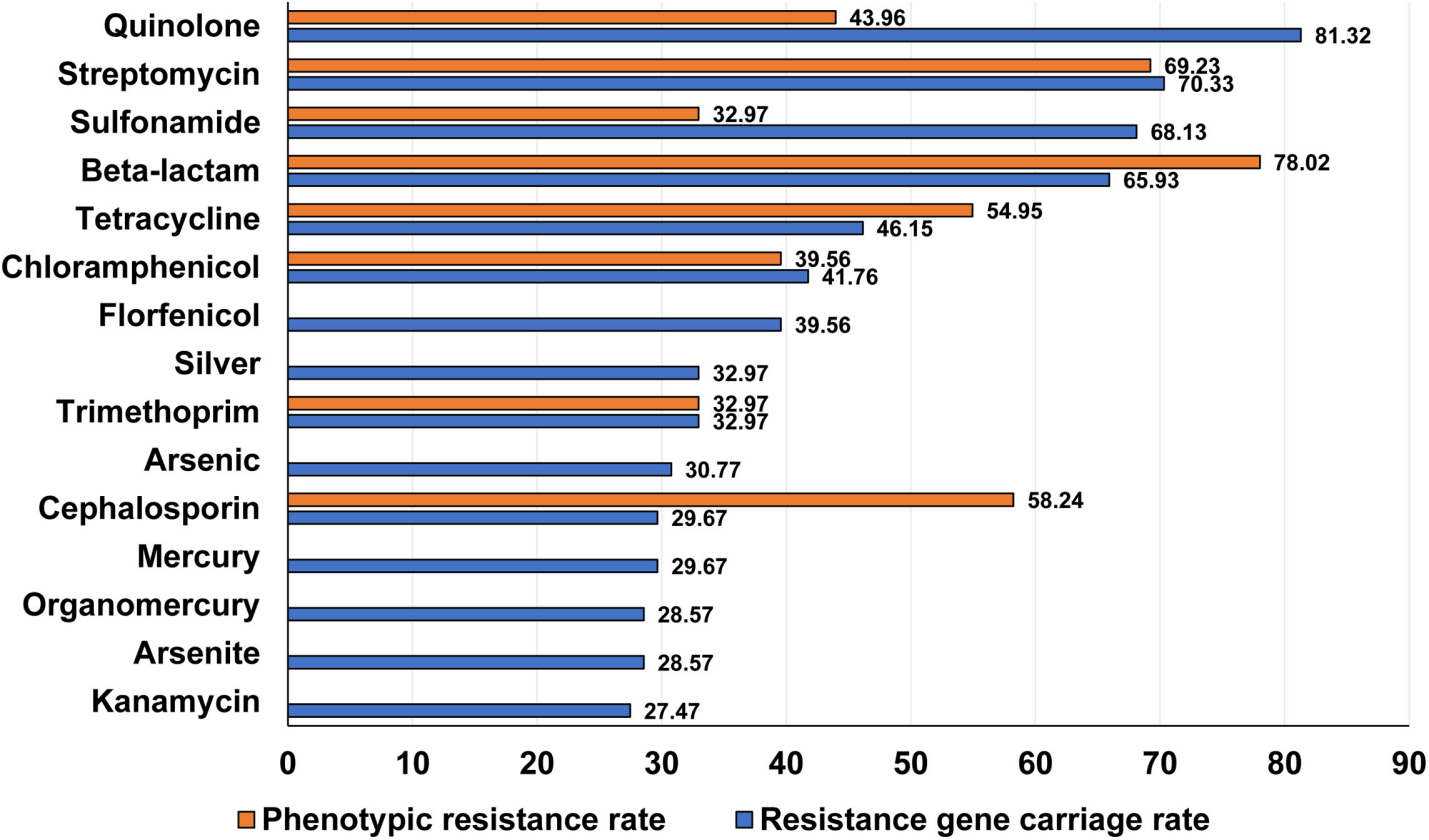
Comparison of phenotypic resistance rates and resistance gene carriage rates in 91 *Salmonella* strains. The bar chart shows the percentage of phenotypic resistance (dark red) and resistance gene carriage (blue) for 15 antibiotics. The resistance rates are indicated at the right end of each bar.

To formally test the genotype-phenotype association, Spearman rank correlation analysis was performed between phenotypic resistance rates and resistance gene carriage rates across eight antibiotics with complete paired data. The analysis revealed a weak and statistically non-significant correlation (Spearman ρ = 0.14, *p* > 0.05), indicating that resistance gene carriage alone does not fully predict phenotypic resistance. Specifically, the phenotypic resistance rate was higher than the gene carriage rate for cephalosporins (difference of +28.57%), beta-lactams (+12.09%), and tetracyclines (+8.80%) (Figure 5), suggesting the possible existence of other resistance mechanisms or that the genetic testing failed to comprehensively cover relevant resistance genes. In contrast, the gene carriage rate was higher than the phenotypic resistance rate for quinolones (difference of −37.36%) and sulfonamides (−35.16%), indicating that resistance genes might be in a silent state or unexpressed. The carriage rates of resistance genes for trimethoprim, chloramphenicol, and streptomycin were basically consistent with their phenotypic resistance rates.

### 3.6 Genetic diversity and phylogenetic relationships of *Salmonella* strains based on cgMLST and cgSNP analyses

**FIGURE 6:**
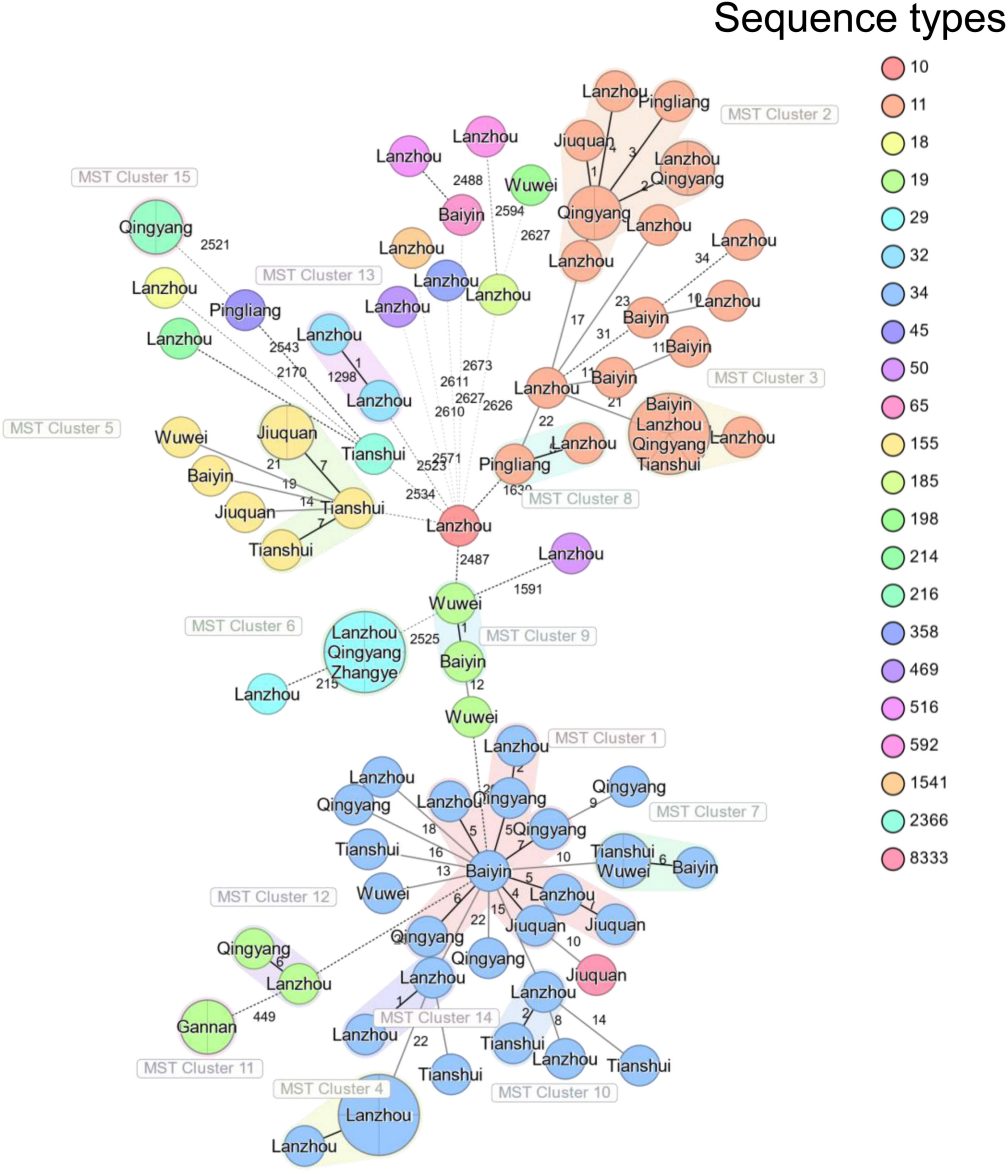
A minimum spanning tree of 91 strains of *Salmonella* based on cgMLST. The tree was constructed using 3002 core genome alleles with a maximum distance threshold of 7. Each circle represents one ST, with circle size proportional to the number of strains. The colors indicate different serogroups. Clusters with closely related strains (allele differences ≤ 7) are highlighted, with Cluster 1 (ST11) and Cluster 2 (ST34) representing the two largest epidemic clones.

The cgMLST clustering analysis was conducted on 91 *Salmonella* strains using 3002 alleles from their core genome. The maximum distance in the MST cluster was set to 7. As illustrated in Figure 6, there were large genetic distances among different ST types, while allele differences within the same ST type were minimal. The strains formed a total of 15 MST clusters. The identification of 15 clusters among 91 strains indicates substantial genetic diversity of *Salmonella* circulating in Gansu Province, suggesting multiple independent introduction events or sustained transmission of distinct lineages.

The 15 clusters revealed distinct population structures. Cluster 1 (ST11, *n* = 9) was the largest, comprising strains from Lanzhou (*n* = 3), Qingyang (*n* = 3), Jiuquan (*n* = 2), and Baiyin (*n* = 1), with core allele differences ranging from 2 to 7. These differences are below the threshold of 10 typically used to define closely related strains, indicating recent clonal expansion and ongoing transmission of ST11 as a major epidemic clone in Gansu Province. Cluster 2 (ST34, *n* = 8) was the second largest, including strains from Lanzhou (*n* = 3), Qingyang (*n* = 3), Pingliang (*n* = 1), and Jiuquan (*n* = 1). The predominance of ST11 and ST34 as the two largest clusters suggests that these are the major epidemic clones driving *Salmonella* infections in Gansu Province in 2024.

Among the remaining clusters, MST Cluster 6 contained identical strains identified as *Salmonella Stanley*, originating from Lanzhou, Qingyang, and Zhangye. In MST Cluster 3, there were 4 identical *S. enterica* strains from Lanzhou, Tianshui, Qingyang, and Baiyin. Additionally, there were 2 monophasic *Salmonella Typhimurium* strains identified from Tianshui and Wuwei (MST Cluster 7), as well as 4 strains from Lanzhou (MST Cluster 4). Furthermore, 2 strains of *S. Typhimurium* from Gannan Prefecture (MST cluster 11) exhibited identical MST loci alleles, indicating a close genetic distance despite originating from different regions.

**FIGURE 7:**
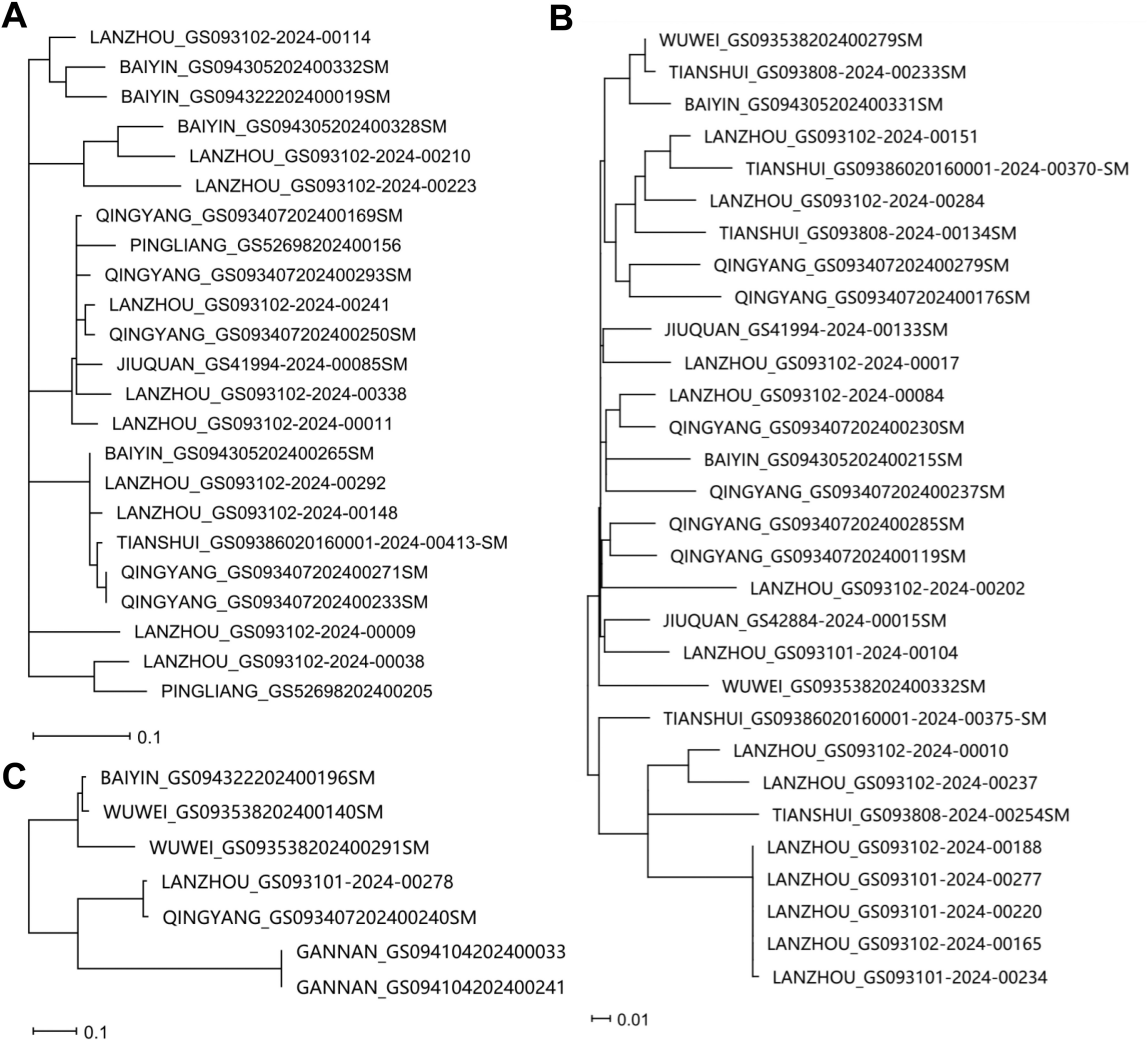
Phylogenetic tree of *Salmonella* based on cgSNP. **(A)** ST11 type *Salmonella*. **(B)** ST34 type *Salmonella*. **(C)** ST19 type *Salmonella*.

Then, we performed cgSNP analysis on the cgMLST-clustered and identical strains to construct a phylogenetic tree. The results revealed that 2 strains from Qingyang had identical SNPs, and 2 strains from Lanzhou and Baiyin were also completely identical within ST11 (Figure 7A). The presence of genetically identical ST11 strains in Lanzhou and Baiyin (approximately 80 km apart), both isolated in August, provides temporal-spatial overlap and suggests a common exposure source and potential inter-regional dissemination of this epidemic clone. In ST34 (Figure 7B), all 4 strains from Lanzhou were identical, while 2 strains from Gannan Prefecture in ST19 (Figure 7C) and 2 strains from Qingyang in ST216 were likewise identical. This suggests that these strains share a common genetic background, indicating a high likelihood of homologous exposure and infection.

**FIGURE 8:**
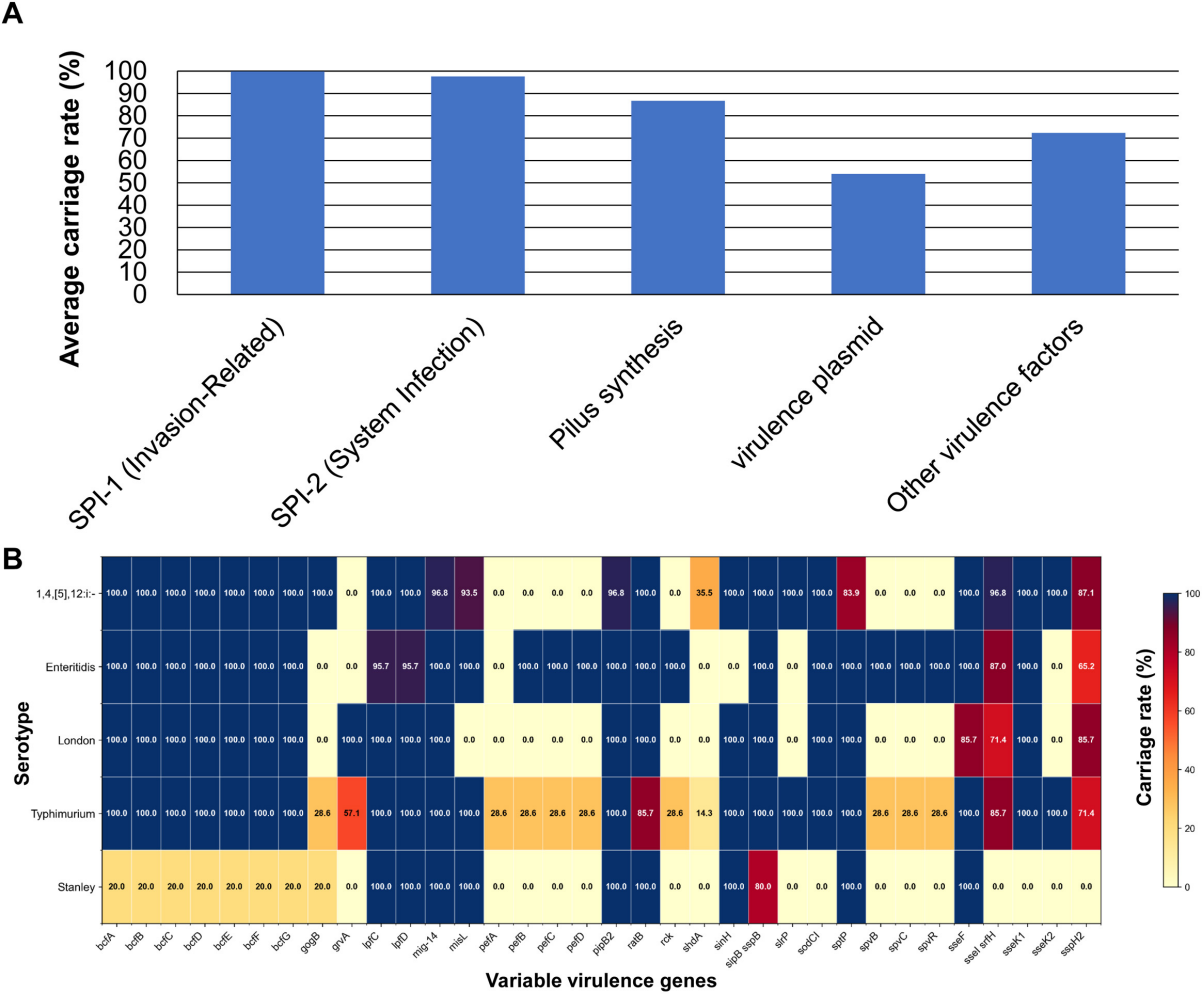
Virulence gene profiles of 91 *Salmonella* strains. **(A)** Average carriage rates of virulence factors. Bars show the carrying rate (%) of virulence genes classified by functional category: SPI-1, SPI-2, pilus synthesis, virulence plasmid, and other virulence factors. **(B)** Heatmap of variable virulence gene carriage rates among the top five serotypes. The heatmap shows the carriage rate (%) of selected variable virulence genes across serotypes 1,4,[5],12:i:–, Enteritidis, Typhimurium, London, and Stanley. Colors represent carriage rates ranging from 0% (light yellow) to 100% (dark blue). Rows indicate serotypes, and columns indicate individual virulence genes.

Using the VFDB database, we predicted 130 virulence genes across the 91 *Salmonella* genomes. A total of 121 effective virulence genes (carried by at least one strain) were identified. Core virulence genes (carried by all 91 strains) were predominantly associated with SPI-1 and SPI-2, which are essential for host cell invasion and intracellular survival, as well as pilus synthesis genes involved in adhesion (Figure 8A). The average carriage rates for SPI-1, SPI-2, and pilus synthesis genes were 98.5%, 96.2%, and 92.7%, respectively, indicating that all strains possess intact *Salmonella* pathogenicity islands conferring foundational pathogenic capacity. In contrast, virulence plasmid-associated genes showed lower average carriage rates (28.5%), and other virulence factors (e.g., *spv*, *pef*, *rck*) exhibited serotype-specific distribution patterns.

Variable virulence genes such as adhesion-related *bcf*, *lpf*, and *csg* genes showed carriage rates ranging from 70% to 99%, suggesting strain-specific differences in biofilm formation and adhesion capacity. Notably, Vi capsule synthesis genes (*tviB/C/D/E*) and transport genes (*vexA/B/C/D/E*) were absent in all strains, confirming their non-typhoidal *Salmonella* status, consistent with the clinical presentation of gastroenteritis rather than typhoidal illness.

Serotype-specific virulence gene signatures were visualized in a heatmap (Figure 8B). For serotype 1,4,[5],12:i:− (*n* = 31), *gogB* (100%) and *slrP* (100%) were specific markers, while the *spv* gene cluster was completely absent (0%). For serotype Enteritidis (*n* = 23), *pefB/C/D* (100%), *rck* (100%), and *spvB/C/R* (100%) were specific markers, whereas *gogB* (0%) and *sinH* (0%) were absent. For serotype Stanley (*n* = 5), the *bcf* gene cluster carriage rate was markedly low (20%), contrasting sharply with other serotypes. For serotype London (*n* = 7), *grvA* (100%) was a specific marker. For serotype Typhimurium (*n* = 7), *slrP* (100%) was present, but *spv* gene cluster carriage was only 28.6%, suggesting possible plasmid loss.

These findings underscore the need for coordinated genomic surveillance across different regions of Gansu Province and warrant further investigation into the food or environmental sources linking geographically distant cases.

## 4 Discussion

The pathogenicity and genomics of *Salmonella* in Gansu Province have been rarely reported. The present study conducted pathogenic and whole-genome characterization analyses on 91 strains of *Salmonella* isolated from diarrheal cases in Gansu Province in 2024. The key findings include: (i) a high multidrug resistance rate of 80.22%, exceeding both national and European averages; (ii) predominance of serotypes 1,4,[5],12:i:− and Enteritidis, consistent with global surveillance trends; and (iii) evidence of potential inter-regional transmission of ST11 and ST34 epidemic clones based on cgSNP analysis.

Geographically, Lanzhou contributed the most isolates. While this may reflect higher population density and greater healthcare access (Liu and Zeng, 2024), it may also indicate sampling bias due to the concentration of sentinel hospitals in urban centers; therefore, the overrepresentation of Lanzhou should not be interpreted as evidence of higher true incidence in this region. Consistent with previous reports (Zheng et al., 2024), children aged 0–9 years were the most affected age group. This may be because children generally have weaker pathogen resistance and insufficient hygiene awareness. Additionally, factors such as communal living in childcare institutions contribute to their increased susceptibility to infectious diarrhea caused by intestinal pathogens (Wang et al., 2024).

Whole-genome sequencing enabled precise serotype prediction, including differentiation of the monophasic variant 1,4,[5],12:i:− from classical Typhimurium (Yang et al., 2014). Using this approach, we identified that monophasic *S. Typhimurium* (1,4,[5],12:i:−) and *Salmonella Enteritidis* were the predominant serotypes. This predominance is consistent with global surveillance data from WHO and CDC, which have identified these serotypes as leading causes of foodborne salmonellosis worldwide (Centers for Disease Control and Prevention, 2025; World Health Organization, 2025a). Furthermore, there was a correlation between serotypes, STs, and serogroups. Our findings are not entirely consistent with observations from slaughterhouse surveillance in Gansu Province, where *S. Typhimurium, Salmonella Derby*, and *Salmonella Rissen* were the main serotypes of *Salmonella* (Ma et al., 2024). This discrepancy likely reflects differences in sampling sources (human clinical cases vs. animal reservoirs) rather than true epidemiological differences, highlighting the need for integrated One Health surveillance. From a One Health perspective, the high prevalence of heavy metal resistance genes (e.g., silver, arsenite) in serotype 1,4,[5],12:i:− — particularly in strains from agricultural regions — raises the hypothesis that agricultural use of heavy metal-containing compounds (e.g., in feed additives or disinfectants) may co-select for antibiotic resistance. This hypothesis is supported by previous studies linking heavy metal and antibiotic resistance co-selection in livestock-associated *Salmonella* (Butaye et al., 2006). On the other hand, our results largely align with the dominant serotypes reported in Yunnan Province (Zhang et al., 2024). Additionally, studies conducted in Jilin Province (Sun et al., 2024) and Hunan Province (Yuan et al., 2024) using traditional serotyping methods also exhibit discrepancies, which may stem from the conventional techniques’ inability to accurately differentiate monophasic *S. Typhimurium* or could relate to regional epidemiological characteristics.

The high resistance rates to β-lactams and aminoglycosides observed in this study are consistent with previous findings (Liu and Zeng, 2024; Yuan et al., 2024; Li et al., 2025). The multidrug resistance rate of 80.22% exceeds the national average reported for non-typhoidal *Salmonella* in China (73.63%) (Chen et al., 2024) and is substantially higher than the 21.4% ciprofloxacin resistance and moderate-to-high levels of ampicillin/tetracycline resistance reported in European surveillance networks (EFSA and ECDC, 2026), suggesting that Gansu Province may have a particularly high burden of multidrug resistant *Salmonella*. The discordance between genotypic and phenotypic resistance for quinolones and sulfonamides warrants further discussion. This may be due to the presence of resistance genes and the expression of resistance phenotypes being influenced by various factors, such as gene expression levels, regulatory mechanisms, and the genetic background of the strains. For example, the resistance mechanism for quinolones is primarily caused by point mutations in the amino acids (gyrA, gyrB, parC) within the QRDR (quinolone resistance-determining region), and *Salmonella* exhibits universal resistance to quinolones only when the strains carry both QRDR mutation genes and plasmid-mediated quinolone resistance genes (Veldman et al., 2011). This genotype-phenotype discordance highlights a critical limitation of relying solely on resistance gene detection for surveillance, a challenge increasingly recognized by global health agencies (World Health Organization, 2025a). Although the resistance rates for naphthidine acid and ciprofloxacin are relatively low, it is essential to monitor their resistance trends and strengthen monitoring and control measures.

The monophasic variant of *S. Typhimurium* isolated from diarrheal cases exhibits multidrug resistance (Deng et al., 2012). The predominance of serotype 1,4,[5],12:i:− in infants under 1 year old is particularly concerning, given its extremely high MDR rate (90.32%). This finding warrants significant attention, particularly regarding the multidrug resistance of *Salmonella* in infants and young children. The resistance analysis results suggest that clinical evaluations should carefully assess the therapeutic value of highly resistant antibiotics, such as ampicillin and streptomycin. In contrast, tigecycline and amikacin, which demonstrate excellent sensitivity rates (≥98%), should be prioritized as treatment options, although their use in pediatric populations requires careful risk-benefit assessment.

The analysis of MLST and SNP based on the core genome facilitates a more precise understanding of genetic variation patterns in *Salmonella* at the genomic level (Kohl et al., 2014). The identification of 15 MST clusters among 91 strains indicates substantial genetic diversity of *Salmonella* circulating in Gansu Province. For strains with a difference of ≤10 alleles in the core genome, a high degree of homology is observed (Zhu et al., 2021). The predominance of ST11 (*S. Enteritidis*) and ST34 (monophasic *S. Typhimurium*) as the two largest clusters suggests that these lineages are actively circulating and may represent the major epidemic clones driving *Salmonella* infections in the region. Furthermore, the presence of strains from different regions within the same clusters indicates close genetic distances across various locales. Notably, two ST11 strains from Lanzhou and Baiyin exhibited identical SNPs and were both isolated in August. This combination of genetic identity, temporal overlap, and geographic proximity strongly suggests a common exposure source. Nevertheless, the specific transmission route—whether through contaminated food supply chains, population movement, or an environmental reservoir—cannot be determined from genomic data alone and requires further epidemiological investigation. Further tracing of the infection sources could aid in preventing the continued spread of this strain. Comparative genomic analysis of strains from different provinces (Liu et al., 2024) may provide early warning signals for strain transmission outbreaks.

The virulence gene profiles further support the pathogenic potential of the isolates. The core virulence gene analysis revealed that all 91 clinical isolates carried intact SPI-1 and SPI-2 pathogenicity islands, which are essential for host cell invasion and intracellular survival (Chen et al., 2016). This finding confirms the pathogenic potential of these strains and is consistent with their isolation from diarrheal cases. The absence of Vi capsule-associated genes across all strains further supports their classification as non-typhoidal *Salmonella*, which typically present with gastroenteritis rather than typhoid fever (Zhang et al., 2025). Serotype-specific virulence gene signatures were observed, with *gogB* and *slrP* uniquely present in serotype 1,4,[5],12:i:− but absent in Enteritidis, while *pef*, *rck*, and *spv* gene clusters were specific to Enteritidis. These signatures may serve as potential molecular markers for serotype identification, as serovar-specific virulence plasmids carry distinct gene repertoires essential for systemic infection (Haneda et al., 2001). The complete absence of the *spv* plasmid-borne virulence cluster in serotype 1,4,[5],12:i:− suggests that this serotype may rely on chromosomal virulence factors rather than plasmid-encoded determinants for pathogenesis.

This study has several limitations. First, the detection rate of *Salmonella* could not be calculated because the total number of diarrheal samples tested across sentinel hospitals was not available from the surveillance system; therefore, our data reflect strain distribution rather than true population-level incidence. Second, the moderate sample size and the reliance on sentinel hospitals may underrepresent rural and remote areas due to unequal access to healthcare. Third, the absence of food, animal, or environmental isolates limits our ability to perform precise source tracing and One Health-based genomic surveillance. Fourth, the genotype-phenotype discordance observed for quinolones and sulfonamides suggests that resistance gene carriage alone does not fully predict phenotypic resistance. Future studies are warranted.

In summary, this study provides the first whole-genome characterization of clinical *Salmonella* isolates from Gansu Province. The alarming level of multidrug resistance and the predominance of serotypes 1,4,[5],12:i:− and Enteritidis underscore the urgent need for enhanced genomic surveillance across northwestern China. From a clinical perspective, empirical use of ampicillin and streptomycin should be reconsidered, while tigecycline and amikacin may serve as alternative options. Future research should prioritize One Health source tracing, mechanistic studies of quinolone resistance (e.g., QRDR mutations), and expanded sampling to rural areas to inform targeted public health interventions.

## Data Availability

The raw data supporting the conclusions of this article will be made available by the authors, without undue reservation. The genome data for the 91 *Salmonella* isolates analyzed in this study have been deposited in the National Microbiology Data Center (NMDC) under accession numbers NMDC60314870 to NMDC60314960. The data can be accessed through the NMDC Genome Database at https://nmdc.cn/resource/genomics/genome by searching the accession numbers. The complete list of isolate accession numbers with direct URLs is provided in Supplementary Table 2.

## Footnotes

1
https://www.bioinformatics.babraham.ac.uk/projects/fastqc

2
https://github.com/ablab/spades

3
http://www.mgc.ac.cn/VFs/

## Supplementary material

Supplementary Table 1 Basic information of 91 Salmonella strains.

Supplementary Table 2 NMDC accession numbers for 91 Salmonella isolates.
